# Transformative Resilience in European Health Governance After COVID-19: A Policy Analysis

**DOI:** 10.3390/healthcare14050569

**Published:** 2026-02-25

**Authors:** Krzysztof Goniewicz, Amir Khorram-Manesh

**Affiliations:** 1 Department of Security Studies, Polish Air Force University, 08-521 Dęblin, Poland; 2 Department of Surgery, Institute of Clinical Sciences, Sahlgrenska Academy, Gothenburg University, 405 30 Gothenburg, Sweden; 3 Disaster Medicine Center, University of Gothenburg, 405 30 Gothenburg, Sweden; 4 Gothenburg Emergency Medicine Research Group (GEMREG), Sahlgrenska Academy, Gothenburg University, 405 30 Gothenburg, Sweden

**Keywords:** European Health Union, health security, transformative resilience, EU governance, European Health Data Space, artificial intelligence, digital sovereignty, strategic preparedness, public health policy

## Abstract

Introduction: The COVID-19 pandemic exposed structural weaknesses in European public health systems while simultaneously accelerating institutional and digital reforms at the European Union (EU) level. This study examines how the EU has evolved from reactive crisis management toward a governance paradigm conceptualized as transformative resilience, understood as the institutional capacity to anticipate, adapt, and structurally reconfigure health governance in response to systemic shocks. Methods: This study employs a structured qualitative policy analysis based on a purposive corpus of key EU legislative and strategic documents (2020–2025), complemented by a contextual review of selected EU-level indicators. The analysis focuses on reforms associated with the European Health Union, including the establishment of the Health Emergency Preparedness and Response Authority (HERA), the development of the European Health Data Space (EHDS), and the adoption of the Artificial Intelligence Act. Results: The findings indicate progressive consolidation of supranational coordination mechanisms, deeper integration of digital infrastructure into health governance, and strategic incorporation of health security into the EU’s broader security architecture. Rather than assessing policy effectiveness, the analysis documents a structural and regulatory shift toward anticipatory and embedded preparedness. Persistent challenges remain, including uneven implementation capacity across member states, disparities in digital maturity, and tensions between innovation and data protection. Conclusions: The EU’s post-pandemic trajectory reflects a distinctive governance model in which health security, digital sovereignty, and democratic safeguards are framed as mutually reinforcing dimensions of resilience within an increasingly complex risk environment.

## 1. Introduction

The COVID-19 pandemic represented not only an unprecedented global health emergency but also a profound geopolitical and institutional turning point that prompted a more proactive response from the European Union [1]. In the early months of the crisis, the fragmentation of national responses, shortages of medical equipment, and disruptions to supply chains exposed the structural limits of European integration in the field of public health [2]. Yet, in its aftermath, the pandemic became a catalyst for systemic reform [3]. What began as an ad hoc coordination effort has evolved into a sustained political project aimed at strengthening collective preparedness, strategic autonomy, and technological sovereignty. In this sense, COVID-19 served as a stress test of the Union’s resilience and, simultaneously, as an accelerator of its transformation.

Earlier research traced this evolution in two distinct phases. The first, published in 2020, examined the European Union’s immediate crisis response, its emergency mechanisms, coordination challenges, and the rapid adaptation of governance structures under pressure [4]. The second, published in 2023, analyzed the subsequent phase of recovery and social resilience, emphasizing economic reconstruction, solidarity mechanisms, and the institutional learning that followed [5]. Building on this empirical and conceptual foundation, the present study turns to the next stage in the European trajectory, which is the consolidation of health governance into a permanent system of prevention, preparedness, and digital integration.

Over the past five years, the EU has initiated a series of far-reaching reforms that collectively constitute the framework of a European Health Union (EHU) [6]. These reforms include the establishment of the Health Emergency Preparedness and Response Authority (HERA) [7], the expansion of the mandates of the European Centre for Disease Prevention and Control (ECDC) [8] and the European Medicines Agency (EMA) [9], the creation of the European Health Data Space (EHDS) [10], and the development of a comprehensive regulatory environment for artificial intelligence (AI). Together, these initiatives mark a decisive shift from reactive crisis management toward anticipatory and data-driven governance, embedding health within the Union’s broader agenda of strategic autonomy and security.

Despite the growing body of literature on European crisis management, few studies have systematically analyzed the institutional and digital transformation underpinning this new governance paradigm. Most existing analyses remain descriptive, focusing on individual policy instruments rather than on the integrated architecture that is now emerging [11,12,13,14,15]. The transformation of the European Health Union, spanning institutional, technological, and ethical dimensions, has yet to be fully conceptualized as a coherent model of twenty-first-century governance [16]. This analytical gap is particularly salient at a time when Europe seeks to define its global role amid accelerating technological competition and complex transnational threats.

Accordingly, this study aims to examine how the European Union has transitioned from resilience-building to structural transformation in health governance. Specifically, it investigates the institutional consolidation of the European Health Union, the development of digital sovereignty through the EHDS and Artificial Intelligence Act (AI Act) [17], and the integration of health security within the EU’s Strategic Compass [18]. By combining systematic policy analysis with contextual review of selected EU-level indicators, the paper seeks to elucidate how these developments are reshaping the foundations of European and global health security. The ultimate objective is to conceptualize this evolution as the emergence of a new governance paradigm, one that redefines resilience not as recovery from crisis, but as the institutional capacity to anticipate, adapt, and act autonomously in the face of future uncertainty.

In this study, transformative resilience is conceptualized analytically as a governance capacity rather than a fixed outcome. It refers to the structured ability of institutional systems to reconfigure their legal, organizational, and technological architecture in response to systemic shocks, thereby enabling anticipatory and adaptive action over time. It is therefore understood as an ongoing process embedded within governance structures, rather than as a measurable end-state.

## 2. Methods

### 2.1. Study Design and Rationale

This study adopts a structured qualitative policy analysis combining systematic document analysis and contextual descriptive indicator review. The purpose was to examine how the European Health Security Framework [19] evolved in the aftermath of the COVID-19 pandemic, with particular emphasis on institutional reforms, policy instruments, and early-stage implementation dynamics between January 2020 and June 2025. Building on previous analyses of the EU’s emergency response and recovery strategies, this research extends the focus from short-term resilience to long-term preparedness and transformative governance. The guiding assumption was that post-pandemic Europe has entered a new phase of institutional consolidation, in which health security, digital sovereignty, and technological innovation are increasingly interlinked within the broader framework of the European Health Union [6].

### 2.2. Data Sources and Collection

Data collection followed a structured and purposive policy corpus approach. The primary material consisted of key official policy and strategic documents issued by the European Commission, the European Parliament, the Council of the European Union, HERA, the ECDC, and the World Health Organization Regional Office for Europe (WHO-EURO). Rather than conducting a comprehensive systematic search of all available materials, the selection focused on institutionally authoritative documents directly shaping post-pandemic health governance reforms between January 2020 and June 2025.

Documents were identified through institutional repositories (EUR-Lex, Europa.eu), legislative databases, and targeted keyword searches (e.g., European Health Union, HERA, pandemic preparedness, resilience, public health security, digital health data). Selection was guided by relevance to the research objectives and substantive impact on EU-level health governance architecture. The analytical corpus comprised approximately 30 core legislative and strategic documents cited throughout the Results Section.

To complement the documentary analysis, selected EU-level statistical indicators were consulted to provide contextual background using data from Eurostat [20], ECDC [8], WHO-EURO [21], and the Organisation for Economic Co-operation and Development (OECD) [22]. These datasets provided measures of health system capacity, expenditure, surveillance performance, digitalization, and public health workforce resilience. Indicators included health expenditure as a percentage of GDP, public health workforce density per 100,000 population, cross-border surveillance reporting indicators, and selected digitalization metrics available at the EU-27 level. The data were harmonized and aggregated at the EU-27 level to ensure comparability across member states. Selected EU-level indicators were reviewed descriptively to contextualize institutional developments. The specific indicators consulted for the contextual descriptive review, along with their definitions and sources, are summarized in Table 1. Additionally, a selective review of peer-reviewed studies and policy briefs from 2020–2025 was performed using Scopus and PubMed to contextualize institutional developments with relevant academic perspectives. The integration of policy analysis with selected contextual indicators supported interpretation of institutional reform trajectories within the broader EU-level preparedness context.

### 2.3. Inclusion and Exclusion Criteria

The analysis included only official and publicly accessible documents issued by recognized European or international institutions. Draft communications, press releases, and non-official commentaries were excluded to maintain analytical consistency and ensure reliability. Documents were selected based on relevance to the research objectives and direct reference to post-pandemic preparedness, resilience, or digital governance.

### 2.4. Analytical Approach

The analytical process combined inductive and deductive reasoning within a thematic synthesis framework. In the first phase, an inductive coding strategy was used to identify emerging themes across the policy corpus, including governance reforms, data interoperability, joint procurement, and funding mechanisms. In the second phase, deductive categorization was applied using a conceptual framework structured around three dimensions: institutional resilience, operational preparedness, and digital and data resilience. This enabled a systematic comparison of the evolving components of the European Health Security Framework.

Document selection and thematic coding were conducted by the authors through iterative analytical review. Discrepancies in interpretation were resolved through consensus discussion to ensure analytical coherence and conceptual consistency.

**Figure 1:**
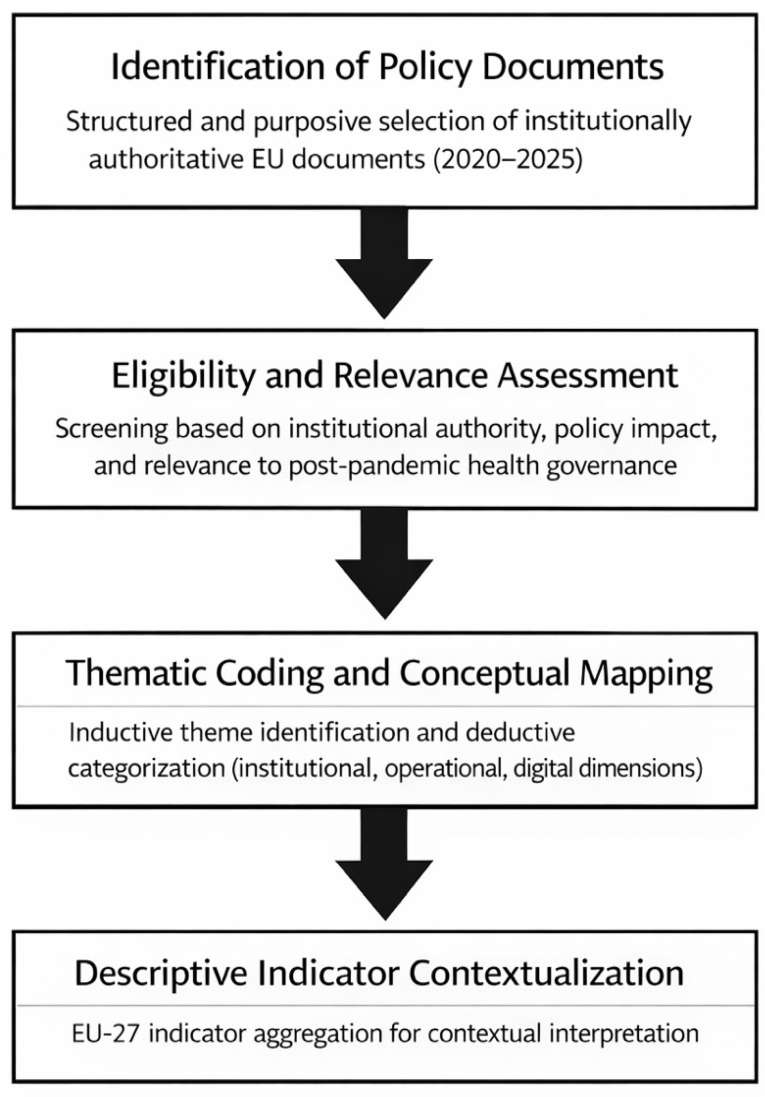
Document selection and analytical workflow of the policy analysis.

Qualitative findings were contextualized using selected EU-level indicators to enhance interpretive robustness. The overall document selection and analytical workflow applied in this study is summarized in Figure 1.

To strengthen analytical precision, this study explicitly differentiated between policy intentions (strategic objectives, normative commitments, and legislative design) and implementation outcomes (institutional operationalization, budgetary commitments, deployment of mechanisms, and observable performance indicators where available). This distinction guided the coding framework and interpretation of findings, ensuring that regulatory ambition was not conflated with demonstrated effectiveness. Given the early implementation stage of several reforms examined, the analysis prioritizes institutional and structural transformation over definitive performance evaluation.

### 2.5. Ethical Considerations

This study was based exclusively on publicly accessible documents and open datasets and did not involve human subjects. Therefore, ethical approval was not required. Nevertheless, the research adhered to principles of transparency, reproducibility, and responsible data use, including full citation of all primary sources and compliance with the General Data Protection Regulation (GDPR) where applicable. The study was conducted in accordance with the ethical principles of the Declaration of Helsinki.

## 3. Results

This section presents the main findings of the analysis, organized around four thematic dimensions: institutional consolidation, digital sovereignty, strategic integration of health security, and ethical governance. Each dimension is examined with attention to the key challenges encountered, the measures adopted, and their systemic implications.

**Figure 2:**
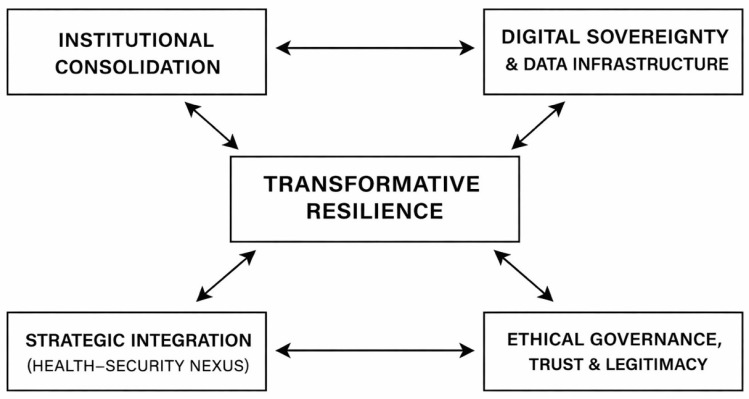
Conceptual model of the four thematic domains of transformative resilience in EU health security governance.

To facilitate interpretation of the findings, Figure 2 provides an integrative overview of the four thematic dimensions identified in the analysis and their contribution to the emergence of transformative resilience in EU health security governance. The figure illustrates how institutional consolidation, digital sovereignty, strategic integration of health security, and ethical governance operate as mutually reinforcing components rather than isolated policy domains.

To further structure the presentation of results, Table 2 maps each of the four thematic dimensions to the principal policy instruments, governance levels, and systemic implications identified in the analysis. Rather than summarizing outcomes, the table serves as a structured reference framework that situates the subsequent sections (Section 3.1, Section 3.2, Section 3.3 and Section 3.4) within the broader architecture of EU health security governance.

### 3.1. Institutional Consolidation: Toward the European Health Union

The transformation of European health governance following the pandemic can be understood through three interrelated aspects: the challenges that exposed systemic fragmentation, the institutional measures adopted to address them, and the broader implications of these reforms for the Union’s health sovereignty.

The post-pandemic period has witnessed the most profound restructuring of health governance in the history of the European Union [4,23]. What began as a crisis-driven response to systemic fragmentation has evolved into a deliberate process of institutional consolidation under the emerging framework of the EHU [24]. This transformation signifies not only a formal strengthening of public health capacities but also a conceptual redefinition of health as a strategic domain of European integration.

At the core of this process lies the establishment of the Health Emergency Preparedness and HERA in 2021, which has become the principal coordinating mechanism for medical countermeasures, supply chain security, and emergency response [7]. HERA’s mandate extends beyond reactive crisis management, as it operationalizes anticipatory surveillance, joint procurement of vaccines and therapeutics, and the development of strategic stockpiles of critical medical supplies. Its structure, integrated within the European Commission yet designed to operate with technical autonomy, reflects a hybrid governance model balancing supranational coordination with national accountability [7]. The authority’s ability to mobilize resources rapidly during subsequent cross-border threats, such as the 2022 monkeypox outbreak and post-pandemic vaccine procurement, illustrates the expanding operational role of HERA during subsequent cross-border health threats.

Parallel to the creation of HERA, both the European ECDC and the EMA have undergone substantial mandate extensions. The ECDC has been granted expanded powers in epidemiological surveillance, risk assessment, and data interoperability across member states, facilitating near–real-time health intelligence [8]. Similarly, the EMA’s strengthened role in monitoring supply chain disruptions and coordinating clinical trials across the EU addresses long-standing inefficiencies exposed during the early COVID-19 response [9]. Together, these reforms create a triadic architecture, HERA, ECDC, and EMA, designed to ensure continuity between preparedness, prevention, and response.

This institutional consolidation represents a marked shift in the EU’s constitutional understanding of health. While health policy remains a shared competence under Article 168 of the Treaty on the Functioning of the European Union [25], the pandemic catalyzed a political consensus that health security constitutes a collective European public good. As a result, the European Health Union functions as a meta-framework that integrates health into the Union’s broader agenda of security, innovation, and social cohesion [26]. Health is thus no longer treated merely as an ancillary component of welfare policy but as a strategic pillar of European sovereignty and resilience. The evolution of legal instruments, funding mechanisms, and cross-sectoral partnerships confirms that the EU has entered a new phase of governance, one in which institutional interdependence and strategic foresight define the parameters of public health capacity [27].

The consolidation achieved through these reforms has begun to address the coordination deficits that hampered the early pandemic response [16,28]. However, it also raises questions regarding the balance of competences between EU institutions and member states. The growing supranationalization of health governance implies a gradual reconfiguration of authority in areas traditionally reserved for national administrations. While this process enhances collective preparedness, it simultaneously necessitates new mechanisms of accountability, transparency, and intergovernmental trust. In this sense, the European Health Union remains an evolving experiment, one that embodies both the lessons of crisis and the ambitions of a continent seeking to institutionalize resilience as a permanent state of governance.

### 3.2. Digital Sovereignty and Data-Driven Health Governance

The evolution of Europe’s digital health governance can be interpreted through the challenges exposed by the pandemic, the regulatory and technological instruments designed to overcome them, and the broader implications of these initiatives for the Union’s pursuit of digital sovereignty.

Parallel to institutional consolidation, the European Union has embarked on a transformative agenda aimed at establishing digital sovereignty as a foundational principle of its health governance architecture [29]. The experience of COVID-19 underscored the critical role of real-time data exchange, interoperable systems, and trustworthy digital infrastructure in effective crisis management [30]. Yet it also revealed the EU’s dependency on non-European cloud services, fragmented national databases, and limited capacity for predictive analytics. In response, the Union has sought to build a cohesive digital ecosystem capable of supporting both health security and technological autonomy [31].

The most ambitious step in this direction is the creation of the EHDS, a regulatory and operational framework designed to facilitate cross-border use of health data for healthcare delivery, research, innovation, and policymaking. Introduced as a legislative proposal in 2022 and subsequently adopted at EU level, with phased implementation foreseen from 2025 onwards, the EHDS represents a paradigm shift from data protection to data empowerment. It establishes common technical standards, interoperable infrastructures, and governance mechanisms enabling citizens, researchers, and public authorities to securely access and exchange health information across member states [10]. In doing so, the EHDS embodies the principle that data mobility is a public good, provided it operates within the safeguards of transparency, consent, and accountability [32]. Beyond its technical function, it also symbolizes a deeper political objective, which is the assertion of Europe’s digital self-determination in the domain of health.

Complementing the EHDS, the AI Act introduces a comprehensive regulatory framework for the design, deployment, and oversight of AI systems, including those used in health-related applications. By classifying health AI tools, such as diagnostic algorithms, triage systems, and clinical decision-support models, as “high-risk,” the Act mandates strict requirements for transparency, human oversight, and post-deployment monitoring [17]. This legal architecture reflects the EU’s normative approach to technology governance, emphasizing that innovation must coexist with ethical safeguards and democratic accountability [17]. In contrast to unregulated markets, Europe positions itself as a “standard-setting power” that defines global norms for trustworthy and human-centric artificial intelligence.

Technological integration is further advanced through the incorporation of interoperable analytics and predictive modeling into preparedness frameworks. Initiatives under the Digital Europe Programme [33] and Horizon Europe [34] have funded the development of digital twins, early warning systems, and real-time epidemiological dashboards that simulate disease dynamics, resource allocation, and supply chain vulnerabilities. These tools allow policymakers to test hypothetical scenarios and optimize responses before crises escalate, marking a transition from reactive management to anticipatory governance. The fusion of digital infrastructure and health security thus enables a new model of decision-making, one grounded in continuous situational awareness and evidence-based forecasting [35].

At the strategic level, the pursuit of digital sovereignty also serves a geopolitical function. By investing in European data infrastructure, cloud computing, and cybersecurity capacities, the EU seeks to reduce reliance on foreign platforms, particularly U.S.-based cloud providers and Chinese AI ecosystems. This effort aligns with the broader agenda of technological autonomy articulated in the EU’s 2020 Digital Strategy [36] and reaffirmed in subsequent Council Conclusions [37]. In this context, health data governance becomes both a matter of privacy and of strategic security. The capacity to store, process, and analyze sensitive health information within Europe’s jurisdiction is increasingly regarded as a prerequisite for safeguarding the Union’s political independence and citizens’ trust [38].

Taken together, these developments indicate a profound transformation in how the EU conceptualizes and manages digital health governance. Data is no longer perceived merely as an administrative by-product but as a strategic asset underpinning resilience, innovation, and sovereignty. Through the combined implementation of the EHDS, the AI Act, and predictive digital infrastructures, the Union is constructing an integrated ecosystem that aspires to make Europe not only digitally capable but digitally sovereign in matters of health and security.

### 3.3. Health Security as Part of the EU Strategic Compass

The evolution of Europe’s security doctrine can be analyzed through the convergence of health and defense policies, the institutional mechanisms established to operationalize this linkage, and the wider implications of integrating health resilience into collective security frameworks.

In the aftermath of COVID-19, the European Union began to reconceptualize health security as a central component of its broader security doctrine [39]. The pandemic revealed that biological hazards, whether natural, accidental, or deliberate, can destabilize societies as profoundly as conventional military threats [40]. This recognition prompted a strategic realignment in which public health resilience is no longer viewed as a purely civilian responsibility but as an integral dimension of European collective security. The publication of the EU Strategic Compass for Security and Defence in 2022 codified this shift, embedding pandemic preparedness, biosecurity, and hybrid threat resilience within the Union’s long-term strategic vision [19].

The integration of pandemics into hybrid threat frameworks marks a conceptual innovation in EU security policy. Hybrid threats, traditionally understood as combinations of cyberattacks, disinformation campaigns, and critical infrastructure sabotage, now explicitly encompass biological and health-related disruptions [41]. The pandemic demonstrated that disinformation surrounding vaccination, the manipulation of medical supply chains, and cyber intrusions into healthcare systems can amplify the societal impact of health crises [42]. Consequently, the EU’s Hybrid Fusion Cell [43] and the European Centre of Excellence for Countering Hybrid Threats [44] have expanded their analytical mandates to include public health vulnerabilities, recognizing that hybrid warfare increasingly operates through the exploitation of social and biological fragility.

This strategic evolution has also deepened the linkage between health and defense under the EU Security Union Strategy [45]. Health systems are now regarded as part of Europe’s critical infrastructure, subject to the same resilience standards as energy, transport, and digital networks. Civil protection and defense planning have become interdependent, with the EU Civil Protection Mechanism [46] serving as a bridge between humanitarian response and strategic preparedness. The inclusion of medical logistics, surveillance, and supply security within defense planning cycles reflects the recognition that pandemics can erode military readiness, disrupt transnational mobility, and undermine societal cohesion, all of which have direct security implications. As a result, the boundaries between “health” and “security” governance are increasingly porous, converging under a unified resilience framework [47].

A significant manifestation of this integration is the growing collaboration with North Atlantic Treaty Organization (NATO) and the European Defence Agency on issues of dual-use logistics and biosecurity [48]. Joint exercises and information-sharing protocols have been developed to coordinate strategic airlift capacities, medical evacuation, and the protection of critical supply chains [48]. These initiatives underscore a pragmatic recognition that in future crises, whether biological, chemical, or hybrid, civilian and military actors will need to operate within a shared operational space [48]. The COVID-19 experience demonstrated the value of military logistics in vaccine distribution and field hospital deployment, while also exposing the institutional silos that hindered rapid coordination [49]. Recent EU–NATO communiqués emphasize the need for interoperability not only in defense technologies but also in the domains of health intelligence, medical countermeasure production, and early warning systems [50,51].

The incorporation of health security into Europe’s strategic compass thus represents a decisive step toward a comprehensive security paradigm, in which the protection of life, infrastructure, and stability are treated as mutually reinforcing objectives [52]. This multidimensional understanding redefines resilience as a strategic capability, anchored in both health systems and defense institutions, and positions the European Union as a normative leader in framing health as a matter of collective security and shared sovereignty. However, as these frameworks mature, they also raise important governance questions regarding accountability, civilian oversight, and the ethical use of security instruments in public health. Balancing efficiency and democratic legitimacy will therefore be critical to ensuring that Europe’s pursuit of health security strengthens, rather than undermines, the social trust on which its resilience ultimately depends.

### 3.4. Ethical Governance, Privacy, and Trust

The evolution of the European Health Union’s digital dimension can be assessed through the ethical dilemmas it generates, the governance mechanisms introduced to address them, and the societal implications for trust, legitimacy, and democratic accountability.

As the European Union advances toward a data-driven model of health governance, questions of ethics, privacy, and public trust have become pivotal to the legitimacy and sustainability of this transformation. The unprecedented expansion of digital infrastructures, surveillance capacities, and data-sharing mechanisms following the pandemic has brought to the forefront the enduring tension between collective security and individual rights [53]. While data integration promises efficiency, foresight, and resilience, it simultaneously exposes vulnerabilities related to privacy, consent, and algorithmic accountability [54]. The management of these tensions will determine whether Europe’s emerging digital health architecture is perceived as a tool of empowerment or as an instrument of control.

The tension between data-driven governance and citizen privacy is intrinsic to the European Health Union’s digital evolution [55]. Systems such as the EHDS and cross-border epidemiological networks rely on large-scale data aggregation to enable predictive modeling and coordinated action. Yet these same systems challenge traditional notions of informational self-determination [10]. The European Union’s regulatory framework, anchored in the GDPR, seeks to reconcile these competing imperatives by emphasizing proportionality, purpose limitation, and informed consent [56]. However, the complexity of real-time data use in health crises often tests the boundaries of these principles, revealing a gap between formal legal safeguards and practical implementation. The EU’s commitment to ethical governance therefore extends beyond compliance, it requires a cultural shift toward transparent stewardship of data as a public trust.

Public acceptance of AI and data sharing has emerged as a decisive factor in the successful implementation of digital health initiatives [57]. Studies across member states consistently demonstrate that citizens’ willingness to share personal health data depends on the perceived fairness, security, and social value of data use [58]. The introduction of the AI Act reflects an acknowledgment that legitimacy in the digital age cannot be sustained by technical competence alone, it must be grounded in moral credibility [17]. Transparent algorithms, human oversight, and explainability are no longer optional design features, they are democratic necessities. Without sustained public confidence, even the most advanced systems risk rejection or underutilization. As such, trust functions not merely as an ethical aspiration but as a form of resilience in itself, a stabilizing force in the governance of uncertainty.

To foster this trust, the European Union has launched a series of initiatives for public engagement and transparency. Mechanisms such as the European Open Data Portal [59], citizen panels under the Conference on the Future of Europe [60], and participatory foresight programs sponsored by Horizon Europe [34] have been instrumental in bringing ethical deliberation into the policy cycle. These initiatives recognize that legitimacy in data governance arises from inclusion and dialogue, not merely from compliance and regulation. By integrating citizen perspectives into the design and oversight of digital infrastructures, the EU seeks to cultivate a sense of shared ownership over collective health intelligence. This participatory model stands in deliberate contrast to more technocratic or commercially driven approaches adopted elsewhere, positioning Europe as a normative innovator in the ethics of digital governance.

Ultimately, the sustainability of the European Health Union will depend as much on the moral architecture of its governance as on its technical capacity. Ethical integrity, transparency, and citizen trust constitute the social capital of resilience. In this respect, Europe’s digital transformation in health represents not only a technological or institutional evolution but also a test of democratic maturity, an experiment in reconciling the imperatives of security and solidarity in a datafied society [61]. The challenge is not simply to regulate innovation but to humanize it, ensuring that the pursuit of collective safety continues to serve, rather than subsume, the dignity and autonomy of the individual.

### 3.5. Synthesis: From Crisis Response to Transformative Resilience

**Figure 3:**
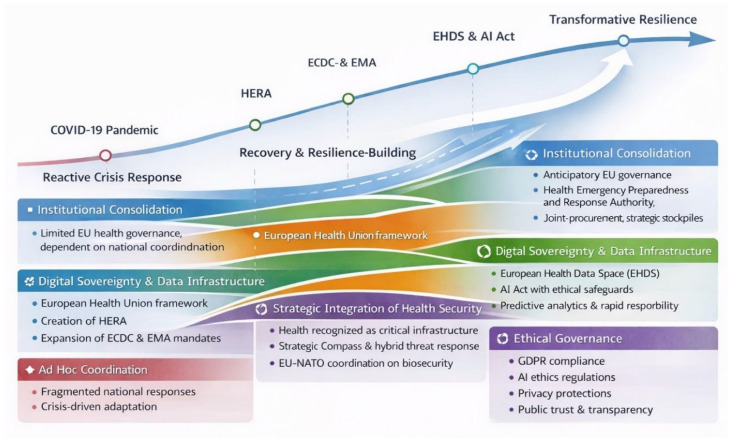
Evolutionary trajectory of EU health security governance toward transformative resilience (2020–2025).

Taken together, the four analytical dimensions examined in Section 3.1, Section 3.2, Section 3.3 and Section 3.4 converge into a coherent evolutionary trajectory of European Union health security governance. This trajectory, summarized in Figure 3, illustrates how the EU has progressed from ad hoc crisis response toward a structurally embedded and integrated governance framework between 2020 and 2025.

At the initial stage, the COVID-19 pandemic exposed deep institutional fragmentation and limited coordination capacities, highlighting the constraints of a governance model primarily oriented toward national responsibility and reactive intervention. The subsequent establishment and strengthening of supranational mechanisms, most notably through the European Health Union framework and the creation of HERA, marked a shift toward institutional consolidation. These developments supported greater continuity between preparedness, prevention, and response and enabled collective action beyond emergency improvisation.

In parallel, the expansion of digital infrastructures and regulatory frameworks introduced a second axis of transformation. The development of the European Health Data Space and the adoption of the Artificial Intelligence Act operationalized data-driven governance while embedding legal and ethical safeguards. This phase corresponds to a transition from fragmented digital initiatives to more coordinated and anticipatory systems capable of supporting surveillance, predictive analytics, and cross-border decision-making.

A third dimension of this trajectory concerns the strategic integration of health security within the EU’s broader security architecture. The incorporation of pandemics and biological threats into the Strategic Compass and related hybrid threat frameworks reframed health systems as critical infrastructure and situated public health preparedness within collective security planning. This convergence of civilian and security domains extended the scope of resilience beyond the health sector to include defense, civil protection, and strategic coordination mechanisms.

Finally, the ethical and societal dimension operates as a cross-cutting condition shaping the legitimacy and acceptability of institutional and technological reforms. The emphasis on privacy, transparency, and citizen trust influenced the design and governance of data-driven systems and contributed to their societal uptake and long-term functionality.

As depicted in Figure 3, these dimensions do not evolve in a strictly sequential manner but interact dynamically over time. Institutional consolidation supports digital integration, while digital capacity reinforces strategic preparedness, and ethical governance shapes trust across all domains. Together, these interacting elements form an integrated architecture of EU health security governance that underpins the transition observed during the study period.

## 4. Discussion

The synthesis presented in Figure 3 indicates that the European Union’s post-pandemic reforms amount to more than incremental policy change. Rather than representing a linear extension of crisis management or recovery-oriented measures, the observed developments point to a qualitative reconfiguration of governance. The institutional, digital, and strategic reforms examined in this study collectively suggest the emergence of a governance logic that can be conceptualized as transformative resilience. In this sense, resilience is understood not merely as adaptive capacity, but as the ability to redesign institutional structures, normative frameworks, and technological infrastructures in anticipation of future systemic risks.

A critical distinction must be drawn between the European Union’s strategic ambitions and the measurable outcomes of reform. Many of the initiatives examined in this study, including the European Health Data Space and elements of the AI Act, are still in early implementation phases. While institutional mandates and regulatory frameworks have been formally established, their operational maturity varies across policy domains and member states. Therefore, the findings should not be interpreted as evidence of fully consolidated performance, but rather as documentation of a structural transition toward a more integrated governance architecture. The transformation observed is primarily institutional and regulatory at this stage, whereas measurable long-term health system outcomes will require future empirical evaluation.

The analysis reveals three overarching dimensions of this transformation. First, institutional continuity and consolidation have emerged as defining features of the European Health Union [6]. The establishment of HERA and the parallel strengthening of ECDC and EMA have bridged previously disconnected domains, public health, economic recovery, and security, into a more cohesive system [7,8,9]. This institutional configuration aims to enhance the Union’s capacity to mobilize collective action and resources more coherently than during the initial phase of the pandemic. The EHDS extends this integration into the digital realm, providing the informational backbone for anticipatory governance and evidence-based decision-making [10]. Together, these reforms signal a shift toward a more permanent infrastructure of preparedness.

Second, the pursuit of technological sovereignty has positioned the EU as a distinctive actor in global health governance [62]. The implementation of the EHDS and the AI Act embodies Europe’s ambition to balance innovation with regulation, fostering digital ecosystems that are competitive yet ethically grounded [10,17]. The EU’s insistence on transparency, accountability, and data protection reflects not a reluctance toward technological progress but an effort to embed democratic principles within it [63]. By asserting control over data flows, algorithmic standards, and digital infrastructures, the Union is redefining autonomy in both functional and geopolitical terms. Digital sovereignty can be interpreted as a potential vector of strategic resilience, ensuring that health data and technological capacity remain under European jurisdiction while contributing to global normative leadership [64].

Third, the EU’s normative power continues to manifest in its capacity to set international standards for ethical and responsible governance. The regulatory frameworks now emerging in Europe, covering AI, data spaces, and cross-border health preparedness, are shaping the global conversation on how digital technologies should serve public goods rather than market imperatives [48,65]. This normative leadership is not purely symbolic, it reflects a broader European philosophy that resilience is inseparable from justice, solidarity, and trust. In a fragmented global order, Europe’s commitment to rule-based governance in the digital and health domains provides a stabilizing counterweight to both techno-authoritarian and laissez-faire models [66].

A brief comparative perspective further contextualizes the distinctiveness of the EU model. In contrast to the United States, where health governance remains highly decentralized and strongly market-oriented, with significant regulatory fragmentation across federal and state levels, the European Union has pursued regulatory harmonization and supranational coordination as central pillars of reform [67]. While the U.S. approach emphasizes institutional flexibility and innovation driven by public–private partnerships, the EU model prioritizes legal standardization, cross-border interoperability, and the embedding of ethical safeguards within regulatory frameworks [66,67]. This divergence reflects different constitutional traditions and political economies. The EU’s trajectory toward transformative resilience is thus characterized less by market-driven adaptation and more by structured regulatory integration and normative governance design.

Despite these achievements, significant challenges remain. The first is the persistent fragmentation between national and supranational competences. Health policy, while now partially integrated, continues to depend on the voluntary coordination of member states [68]. Divergences in administrative capacity, political will, and strategic priorities limit the speed and uniformity of implementation [69]. The second challenge concerns uneven digital capacity across the Union. Variations in data infrastructure, cybersecurity maturity, and human capital risk creating a multi-speed Europe in digital health [70].

Importantly, the degree of implementation and absorptive capacity varies considerably across EU member states. Differences in digital infrastructure, public health workforce density, administrative capacity, and prior investment in e-governance create uneven starting points for reforms such as the EHDS and AI governance frameworks [71]. Western and Northern European member states generally demonstrate higher levels of digital maturity and interoperability readiness, whereas several Central and Eastern European countries face constraints related to infrastructure gaps and workforce shortages [72]. These asymmetries do not negate the integrative ambition of the European Health Union but suggest that transformative resilience may unfold at different speeds across the Union. Consequently, EU-level regulatory harmonization must be accompanied by targeted capacity-building and financial support mechanisms to prevent widening structural divergence.

Finally, the EU faces an enduring tension between innovation and privacy, autonomy and solidarity [73]. Striking the right equilibrium between technological progress and the ethical imperatives of transparency, inclusivity, and individual rights remains a delicate balancing act, particularly as predictive analytics and AI systems become more deeply embedded in governance [74,75].

## 5. Policy Implication

The policy implications of these findings are both strategic and operational. The EU should establish permanent coordination mechanisms linking public health, digital innovation, and security policy under a unified governance framework. Such mechanisms could institutionalize the synergies already evident in HERA, the EHDS, and the Strategic Compass, ensuring that resilience becomes an enduring characteristic of the Union’s policy architecture rather than a transient post-crisis aspiration. Furthermore, digital literacy and health security competencies should be integrated into the EU Civil Protection Mechanism and national preparedness programs to enhance interoperability between health, civil, and defense sectors. Finally, the measurement of resilience itself must evolve. Traditional economic or infrastructural indicators are no longer sufficient, new resilience metrics should capture dimensions such as trust in institutions, data transparency, and the adaptability of governance systems to technological disruption.

Taken together, these insights suggest that the European Union is not merely responding to the lessons of the pandemic but is actively redefining what it means to govern in an age of complexity. Transformative resilience, as conceptualized in this study, is not a static end-state but a dynamic governance capacity operating through institutional redesign, digital integration, and strategic coordination. It represents a distinctly European contribution to the theory and practice of global governance, offering a model in which health security, innovation, and democracy are treated not as competing objectives but as mutually reinforcing foundations of a resilient and forward-looking Union.

To operationalize these recommendations, the EU could integrate resilience metrics and cross-sectoral training modules into upcoming multiannual financial frameworks and the European Semester, linking preparedness goals with fiscal and regulatory incentives. Moreover, Europe’s approach provides a transferable model for global health governance, demonstrating how regional integration can reinforce both autonomy and solidarity in managing transboundary risks [76].

## 6. Limitations

While this study provides a comprehensive examination of the European Union’s post-pandemic transformation in health governance, several limitations should be acknowledged. First, the analysis is based primarily on publicly available policy documents, legislative texts, and open datasets. As such, it does not capture the informal negotiations, political dynamics, and internal deliberations that often shape EU decision-making. These processes, particularly those within the European Council and between member states, remain partially opaque to external observers and could influence the interpretation of institutional evolution.

Second, the study is constrained by the temporal proximity of many of the reforms under review. Key initiatives such as the HERA, the EHDS, and the Artificial Intelligence Act are still in the early stages of implementation. Their long-term effectiveness, interoperability, and integration across member states cannot yet be empirically assessed. As a result, the conclusions presented here should be understood as analytical rather than evaluative, focusing on the structural and conceptual dimensions of transformation rather than measurable outcomes.

Third, the comparative assessment of member states’ capacities faces methodological limitations due to uneven data availability and varying degrees of digital maturity. Despite efforts to harmonize indicators across Eurostat, ECDC, and OECD sources, differences in reporting standards, data granularity, and temporal coverage may affect comparability. Similarly, qualitative interpretations derived from policy analysis inevitably carry an element of subjectivity, even when supported by systematic coding and contextual indicator review.

Finally, as with most document-based research, this study cannot fully account for the broader geopolitical and socio-economic variables influencing EU health governance. External shocks, ranging from energy insecurity to emerging global conflicts, intersect with health and digital policy in complex ways that extend beyond the scope of this paper. Nonetheless, by integrating multiple sources and analytical perspectives, this study provides a robust and coherent understanding of how the European Union is institutionalizing transformative resilience in its health and security systems.

Future research should complement document-based analysis with qualitative inquiry, including semi-structured interviews with health ministers, EU-level policymakers, and senior officials involved in the implementation of the European Health Union. Such comparative research, particularly across Western and Eastern European member states, would allow for a more nuanced understanding of governance dynamics, political negotiation processes, and implementation variability. Integrating elite interviews with institutional analysis could provide deeper insight into how transformative resilience operates in practice beyond formal regulatory design.

## 7. Conclusions

The European Union’s post-pandemic evolution marks a decisive shift from reactive crisis management to anticipatory and integrated governance. The consolidation of institutions under the European Health Union, the establishment of HERA, and the digital architecture shaped by the EHDS and AI Act demonstrate how health, technology, and security have become strategically interconnected. Together, these reforms embody transformative resilience, a governance paradigm that couples institutional stability with adaptive innovation. Sustaining this progress will require continued investment in data interoperability, public trust, and equitable participation across member states. Ultimately, the European Health Union reflects a vision of governance that aligns solidarity, sovereignty, and foresight, positioning Europe as a global leader in health security and digital integrity.

## Figures and Tables

**Table 1:** Selected EU-27 indicators consulted for contextual descriptive review.

Indicator	Operational Definition	Source	Time Coverage	Handling of Missing Data
Health expenditure (% of GDP)	Total current health expenditure as a percentage of gross domestic product	Eurostat [20]	2020–2024	Aggregated using available EU-27 reported values; no imputation applied
Public health workforce density	Number of public health professionals per 100,000 population	WHO-EURO [21]	2020–2024	Based on reported national data; countries with incomplete reporting excluded from year-specific aggregation
Surveillance reporting performance	Timeliness and completeness of infectious disease surveillance reporting	ECDC [8]	2020–2024	EU-level aggregates used where available; descriptive comparison only
Digital health readiness metrics	Selected digital health and interoperability indicators at EU level	OECD [22]/Eurostat [20]	2020–2024	Aggregated at EU-27 level; descriptive contextual review without statistical imputation.

**Table 2:** Mapping of the four thematic domains constituting transformative resilience as a governance capacity.

Thematic Domain	Key EU Policy Instruments and Mechanisms	Governance Level	Systemic Implications
Institutional consolidation	European Health Union framework; Health Emergency Preparedness and Response Authority (HERA); extended mandates of the European Centre for Disease Prevention and Control (ECDC) and the European Medicines Agency (EMA); joint procurement mechanisms	Supranational with shared competences	Institutional continuity; strengthened collective preparedness; partial supranationalisation of health governance; enhanced coordination across member states
Digital sovereignty and data infrastructure	European Health Data Space (EHDS); Artificial Intelligence Act (AI Act); interoperability standards; predictive analytics and early warning systems supported by Digital Europe and Horizon Europe programmes	Supranational and regulatory	Data-driven and anticipatory governance; technological autonomy; improved cross-border data interoperability; strengthened decision-support capacity
Strategic integration (health–security nexus)	EU Strategic Compass for Security and Defence; EU Civil Protection Mechanism; hybrid threat frameworks; EU–NATO cooperation on medical logistics and biosecurity	Cross-sectoral (civil–military)	Recognition of health systems as critical infrastructure; integration of health resilience into security planning; convergence of civilian and defence preparedness
Ethical governance, trust and legitimacy	General Data Protection Regulation (GDPR); ethical safeguards under the AI Act; transparency mechanisms; citizen participation initiatives	Normative and societal	Protection of fundamental rights; public trust in data-driven governance; democratic legitimacy; ethical sustainability of digital and institutional reforms

## Data Availability

The data presented in this study were derived from publicly available policy documents and open-access institutional datasets. All sources are cited within the article. The data analyzed are available from the respective public repositories, including EUR-Lex, the European Commission, Eurostat, ECDC, WHO-EURO, and OECD websites.

## Abbreviations

The following abbreviations are used in this manuscript:

AI	Artificial Intelligence
AI Act	Artificial Intelligence Act
ECDC	European Centre for Disease Prevention and Control
EDA	European Defence Agency
EHDS	European Health Data Space
EHU	European Health Union
EMA	European Medicines Agency
EPRS	European Parliament Research Service
EU	European Union
GDPR	General Data Protection Regulation
HERA	Health Emergency Preparedness and Response Authority
NATO	North Atlantic Treaty Organization
OECD	Organisation for Economic Co-operation and Development
WHO-EURO	World Health Organization Regional Office for Europe

## References

[1] Bergner S. (2023). The role of the European Union in global health: The EU’s self-perception(s) within the COVID-19 pandemic. Health Policy.

[2] Pacces A.M., Weimer M. (2020). From diversity to coordination: A European approach to COVID-19. Eur. J. Risk Regul..

[3] Tuccillo R.J. (2022). The COVID-19 Pandemic Was a Catalyst For Change. J. Gov. Financ. Manag..

[4] Goniewicz K., Khorram-Manesh A., Hertelendy A.J., Goniewicz M., Naylor K., Burkle F.M. (2020). Current response and management decisions of the European Union to the COVID-19 outbreak: A review. Sustainability.

[5] Goniewicz K., Khorram-Manesh A., Burkle F.M., Hertelendy A.J., Goniewicz M. (2023). The European Union’s post-pandemic strategies for public health, economic recovery, and social resilience. Glob. Transit..

[6] European Commission European Health Union. 2025. https://europeanhealthunion.eu.

[7] Commission of the European Union Health Emergency Preparedness and Response Authority (HERA). https://commission.europa.eu/about/departments-and-executive-agencies/health-emergency-preparedness-and-response-authority_en.

[8] European Centre for Disease Prevention and Control (ECDC) https://www.ecdc.europa.eu/en.

[9] European Medicines Agency (EMA) https://www.ema.europa.eu/en/homepage.

[10] European Commission European Health Data Space (EHDS) Regulation. https://health.ec.europa.eu/ehealth-digital-health-and-care/european-health-data-space-regulation-ehds_en.

[11] Ziozias C., Tsagalis A., Anthopoulos L. (2024). Uncovering Organization Emergent Digital Transformation Strategies to deal with Crises: A Review of Bibliometric Analyses and of a European Initiative. J. Sustain. Res..

[12] Boin A., Rhinard M. (2023). Crisis management performance and the European Union: The case of COVID-19. J. Eur. Public Policy.

[13] Trauttmansdorff P., Felt U. (2023). Between infrastructural experimentation and collective imagination: The digital transformation of the EU border regime. Sci. Technol. Hum. Values.

[14] Androniceanu A. (2023). The new trends of digital transformation and artificial intelligence in public administration. Rev. Adm. Si Manag. Public RAMP.

[15] Vărzaru A.A., Bocean C.G. (2024). Digital transformation and innovation: The influence of digital technologies on turnover from innovation activities and types of innovation. Systems.

[16] McKee M., de Ruijter A. (2024). The path to a European health union. Lancet Reg. Health–Eur..

[17] Artificial Intelligence Act. https://artificialintelligenceact.eu.

[18] European External Action Service (EEAS) Strategic Compass for Security and Defence. https://www.eeas.europa.eu/eeas/strategic-compass-security-and-defence-1_en.

[19] European Commission Health Security and Infectious Diseases. https://health.ec.europa.eu/health-security-and-infectious-diseases_en.

[20] Eurostat https://ec.europa.eu/eurostat.

[21] World Health Organization Regional Office for Europe (WHO Europe) https://www.who.int/europe/.

[22] OECD OECD Finds Growing Transparency Efforts Among Leading AI Developers. 2025. https://www.oecd.org/en/about/news/press-releases/2025/09/oecd-finds-growing-transparency-efforts-among-leading-ai-developers.html.

[23] Petr B., Čech T., Frühauf L., Jusić M., Kreisinger J., Reznikow A., Veselý A., Witz P. (2025). Building Capacity for Evidence-Informed Policymaking in Governance and Public Administration in a Post-Pandemic Europe-Country Analysis, Policy Recommendations and Implementation Roadmap Czech Republic.

[24] Brooks E., de Ruijter A., Greer S.L., Rozenblum S. (2023). EU health policy in the aftermath of COVID-19: Neofunctionalism and crisis-driven integration. J. Eur. Public Policy.

[25] European Union Summary of the Treaty on the Functioning of the European Union. https://eur-lex.europa.eu/EN/legal-content/summary/treaty-on-the-functioning-of-the-european-union.html.

[26] Burau V., Mejsner S.B., Falkenbach M., Fehsenfeld M., Kotherová Z., Neri S., Wallenburg I., Kuhlmann E. (2024). Post-COVID health policy responses to healthcare workforce capacities: A comparative analysis of health system resilience in six European countries. Health Policy.

[27] Umbach G. (2024). Futures in EU governance: Anticipatory governance, strategic foresight and EU Better Regulation. Eur. Law J..

[28] Ferrera M., Kyriazi A., Miró J. (2024). Integration through expansive unification: The birth of the European Health Union. Publius J. Fed..

[29] Troitiño D.R., Mazur V., Kerikmäe T. (2024). E-governance and integration in the European union. Internet Things.

[30] Nowotny H. (2025). Foreword: The making of digital Europe. Project Europe: The Making of European Digital Innovation, Policy and Society.

[31] Blancato F.G. (2024). The cloud sovereignty nexus: How the European Union seeks to reverse strategic dependencies in its digital ecosystem. Policy Internet.

[32] Ibáñez L.D., Domingue J., Kirrane S., Seneviratne O., Third A., Vidal M.E. (2023). Trust, accountability, and autonomy in knowledge graph-based AI for self-determination. arXiv.

[33] European Commission The Digital Europe Programme. https://digital-strategy.ec.europa.eu/en/activities/digital-programme.

[34] European Commission Horizon Europe Programme. https://research-and-innovation.ec.europa.eu/funding/funding-opportunities/funding-programmes-and-open-calls/horizon-europe_en.

[35] Huang J., Xu Y., Wang Q., Wang Q.C., Liang X., Wang F., Zhang Z., Wei W., Zhang B., Huang L. (2025). Foundation models and intelligent decision-making: Progress, challenges, and perspectives. Innovation.

[36] EU for Digital EU Digital Strategy. https://eufordigital.eu/discover-eu/eu-digital-strategy/.

[37] Council of the European Union/European Council European Council Conclusions. https://www.consilium.europa.eu/en/european-council/conclusions/.

[38] de Ruijter A., Hervey T., Prainsack B. (2024). Solidarity and trust in European Union health governance: Three ways forward. Lancet Reg. Health–Eur..

[39] Sheng E.L., Nong J., Yin Y. (2025). Responding to COVID-19: Theorizing European Union governance and health policy. J. Contemp. Eur. Stud..

[40] Kaneberg E., Piotrowicz W.D., Abikova J., Listou T., Schiffling S.A., Paciarotti C., Vega D., Adalgeirsdottir K. (2023). Defence organizations in emergency networks: The early response to COVID-19 in Europe. J. Humanit. Logist. Supply Chain. Manag..

[41] Arkan Z. (2025). European Security and Hybrid Threats: A Narrative in the Making.

[42] Goyal N.K. (2025). Weaponizing Misinformation and Cyberattacks on Supply Chains. Mass Media and Impact of Fake News on Supply Chains.

[43] European Commission A Europe that Protects: Good Progress on Tackling Hybrid Threats. https://ec.europa.eu/commission/presscorner/detail/en/ip_19_2788.

[44] European Centre of Excellence for Countering Hybrid Threats. https://www.hybridcoe.fi.

[45] European Commission European Security Union. https://commission.europa.eu/strategy-and-policy/priorities-2019-2024/promoting-our-european-way-life/european-security-union_en.

[46] EU Civil Protection Mechanism. https://civil-protection-humanitarian-aid.ec.europa.eu/what/civil-protection/eu-civil-protection-mechanism_en.

[47] Poroes C., Seematter-Bagnoud L., Wyss K., Peytremann-Bridevaux I. (2023). Health system performance and resilience in times of crisis: An adapted conceptual framework. Int. J. Environ. Res. Public Health.

[48] European Defence Agency (EDA) https://european-union.europa.eu/institutions-law-budget/institutions-and-bodies/search-all-eu-institutions-and-bodies/european-defence-agency-eda_en.

[49] Nylen A.J., Bashir A., Manokaran K., Polatty D., Levine A. (2023). Civilian-Military Coordination During the U.S. National Response to COVID-19.

[50] NATO Allied Command Transformation Enhancing Readiness through Medical Innovation and Interoperability. https://www.act.nato.int/article/milmed-coe-2025/.

[51] European Parliament Research Service (EPRS) EPRS_BRI(2025)772922. https://www.europarl.europa.eu/thinktank/en/document/EPRS_BRI(2025)772922.

[52] Sus M. (2024). Exploring the dynamics of policy change in EU security and defence: Policy entrepreneurs behind the strategic compass. West Eur. Politics.

[53] Małagocka K. (2024). Navigating digital privacy and surveillance: Post-COVID regulatory and theoretical insights. Politics Gov..

[54] Mansoor S.I.U. An Interface Between Digital Privacy and Human Rights: The Challenges Ahead. 2023. https://www.academia.edu/119055682/An_Interface_between_Digital_Privacy_and_Human_Rights_The_Challenges_Ahead.

[55] Aidinlis S. (2025). Governing Digital Public Infrastructure: Innovation, Inclusion and Societal Progress in the Age of AI.

[56] European Union General Data Protection Regulation (GDPR). https://gdpr-info.eu.

[57] Kumar R., Singh A., Subahi A., Humaida M., Joshi S., Sharma M. (2025). Leveraging artificial intelligence to achieve sustainable public healthcare services in Saudi Arabia: A systematic literature review of critical success factors. Comput. Model. Eng. Sci..

[58] Benevento M., Mandarelli G., Carravetta F., Ferorelli D., Caterino C., Nicolì S., Massari A., Solarino B. (2023). Measuring the willingness to share personal health information: A systematic review. Front. Public Health.

[59] European Union Data.europa.eu. https://data.europa.eu/en.

[60] European Parliament European Citizens’ Panels. https://multimedia.europarl.europa.eu/pl/topic/european-citizens-panels_20104.

[61] Calzati S., van Loenen B. (2025). Beyond federated data: A data commoning proposition for the EU’s citizen-centric digital strategy. AI Soc..

[62] Broeders D., Cristiano F., Kaminska M. (2023). In search of digital sovereignty and strategic autonomy: Normative power Europe to the test of its geopolitical ambitions. JCMS J. Common Mark. Stud..

[63] Laux J., Wachter S., Mittelstadt B. (2024). Three pathways for standardisation and ethical disclosure by default under the European Union Artificial Intelligence Act. Comput. Law Secur. Rev..

[64] Hulkó G., Kálmán J., Lapsánszky A. (2025). The politics of digital sovereignty and the European Union’s legislation: Navigating crises. Front. Political Sci..

[65] Gatt A.R., Vella Bonanno P., Zammit R. (2024). Ethical considerations in the regulation and use of herbal medicines in the European Union. Front. Med. Technol..

[66] Ozturk I. (2025). Capitalist Disruptions and the Democratic Retreat: A US–EU–China Comparison. J. Popul. Stud..

[67] Abimbola S., Baatiema L., Bigdeli M. (2019). The impacts of decentralization on health system equity, efficiency and resilience: A realist synthesis of the evidence. Health Policy Plan..

[68] Oliveira R., Santinha G., Sá Marques T. (2023). The Impacts of health decentralization on equity, efficiency, and effectiveness: A scoping review. Sustainability.

[69] Beber C.L., Aragrande M., Canali M. (2025). Policies and strategies to control antimicrobial resistance in livestock production: A comparative analysis of national action plans in European Union Member States. Health Policy.

[70] Zhelyazkova A., Thomann E., Ruffing E., Princen S. (2024). Differentiated policy implementation in the European Union. West Eur. Politics.

[71] Carver J. (2024). More bark than bite? European digital sovereignty discourse and changes to the European Union’s external relations policy. J. Eur. Public Policy.

[72] Sharma D., Gupta S. (2025). The role of e-government in modern public health systems. Startup-Driven E-Government: Digital Innovation for Sustainable Ecosystems.

[73] Becker A., Becker J., Zdziebko T. (2025). Digital Maturity of European Union Enterprises in the Context of Ai Implementation–An Empirical Approach Using Cluster Analysis. Intell. Manag. Artif. Intell. Trends Chall. Oppor..

[74] Saracino D. (2024). Understanding solidarity in the European Union: An analytical framework. Theory Soc..

[75] Goniewicz K., Khorram-Manesh A. (2026). Resilience and Recovery in Disaster and Emergency Management.

[76] Goniewicz K., Burkle F.M., Khorram-Manesh A. (2025). Transforming global public health: Climate collaboration, political challenges, and systemic change. J. Infect. Public Health.

